# Bone Health After Exercise Alone, GLP-1 Receptor Agonist Treatment, or Combination Treatment: A Secondary Analysis of a Randomized Clinical Trial

**DOI:** 10.1001/jamanetworkopen.2024.16775

**Published:** 2024-06-25

**Authors:** Simon Birk Kjær Jensen, Victor Sørensen, Rasmus Michael Sandsdal, Eva Winning Lehmann, Julie Rehné Lundgren, Christian Rimer Juhl, Charlotte Janus, Tummas Ternhamar, Bente Merete Stallknecht, Jens Juul Holst, Niklas Rye Jørgensen, Jens-Erik Beck Jensen, Sten Madsbad, Signe Sørensen Torekov

**Affiliations:** 1 Department of Biomedical Sciences, Faculty of Health and Medical Sciences, University of Copenhagen, Copenhagen, Denmark; 2 Mental Health Centre Glostrup, University of Copenhagen, Glostrup, Denmark; 3 Department of Endocrinology, Hvidovre University Hospital, Copenhagen, Denmark; 4 Novo Nordisk Foundation Center for Basic Metabolic Research, Faculty of Health and Medical Sciences, University of Copenhagen, Copenhagen, Denmark; 5 Department of Clinical Biochemistry, Rigshospitalet, Glostrup, Denmark; 6 Department of Clinical Medicine, University of Copenhagen, Copenhagen, Denmark

## Abstract

**Importance:**

A major concern with weight loss is concomitant bone loss. Exercise and glucagon-like peptide-1 receptor agonists (GLP-1RAs) represent weight loss strategies that may protect bone mass despite weight loss.

**Objective:**

To investigate bone health at clinically relevant sites (hip, spine, and forearm) after diet-induced weight loss followed by a 1-year intervention with exercise, liraglutide, or both combined.

**Design, Setting, and Participants:**

This study was a predefined secondary analysis of a randomized clinical trial conducted between August 2016 and November 2019 at the University of Copenhagen and Hvidovre Hospital in Denmark. Eligible participants included adults aged 18 to 65 years with obesity (body mass index of 32-43) and without diabetes. Data analysis was conducted from March to April 2023, with additional analysis in February 2024 during revision.

**Interventions:**

After an 8-week low-calorie diet (800 kcal/day), participants were randomized to 1 of 4 groups for 52 weeks: a moderate- to vigorous-intensity exercise program (exercise alone), 3.0 mg daily of the GLP-1 RA liraglutide (liraglutide alone), the combination, or placebo.

**Main Outcomes and Measures:**

The primary outcome was change in site-specific bone mineral density (BMD) at the hip, lumbar spine, and distal forearm from before the low-calorie diet to the end of treatment, measured by dual-energy x-ray absorptiometry in the intention-to-treat population.

**Results:**

In total, 195 participants (mean [SD] age, 42.84 [11.87] years; 124 female [64%] and 71 male [36%]; mean [SD] BMI, 37.00 [2.92]) were randomized, with 48 participants in the exercise group, 49 participants in the liraglutide group, 49 participants in the combination group, and 49 participants in the placebo group. The total estimated mean change in weight losses during the study was 7.03 kg (95% CI, 4.25-9.80 kg) in the placebo group, 11.19 kg (95% CI, 8.40-13.99 kg) in the exercise group, 13.74 kg (95% CI, 11.04-16.44 kg) in the liraglutide group, and 16.88 kg (95% CI, 14.23-19.54 kg) in the combination group. In the combination group, BMD was unchanged compared with the placebo group at the hip (mean change, −0.006 g/cm^2^; 95% CI, −0.017 to 0.004 g/cm^2^; *P* = .24) and lumbar spine (−0.010 g/cm^2^; 95% CI, −0.025 to 0.005 g/cm^2^; *P* = .20). Compared with the exercise group, BMD decreased for the liraglutide group at the hip (mean change, −0.013 g/cm^2^; 95% CI, −0.024 to −0.001 g/cm^2^; *P* = .03) and spine (mean change, −0.016 g/cm^2^; 95% CI, −0.032 to −0.001 g/cm^2^; *P* = .04).

**Conclusions and Relevance:**

In this randomized clinical trial, the combination of exercise and GLP-1RA (liraglutide) was the most effective weight loss strategy while preserving bone health. Liraglutide treatment alone reduced BMD at clinically relevant sites more than exercise alone despite similar weight loss.

**Trial Registration:**

EudraCT: 2015-005585-32

## Key Points

**Question:**

Does exercise alone, glucagon-like peptide-1 receptor agonist (GLP-1 RA) treatment, or both treatments combined preserve clinically relevant site-specific bone mineral density (BMD) during weight loss?

**Findings:**

In this secondary analysis of a randomized clinical trial among 195 adults with obesity, the combination of exercise and GLP-1 RA preserved hip, spine, and forearm BMD despite larger weight loss. GLP-1 RA treatment alone reduced hip and spine BMD compared with placebo or exercise alone.

**Meaning:**

These findings suggest that the addition of exercise to GLP-1 RA treatment is an effective weight loss strategy while preserving bone health.

## Teaser

This randomized clinical trial analyzes whether exercise, glucagon-like peptide-1 (GLP-1) receptor agonist treatment, or their combination preserve site-specific bone mineral density for individuals with obesity undergoing weight loss intervention.

## Introduction

Weight loss reduces obesity-related comorbidities.^1^ However, concomitant bone loss typically occurs with weight loss, seen as decreased bone mineral density (BMD) and increased bone turnover.^2^ Low BMD is associated with increased risk of fractures,^3^ and mortality rates increase following any fractures, particularly hip and vertebral fractures, but also other major fractures.^4^ Weight loss–induced bone loss is a particular concern in older adults.^5^ However, bone loss following weight loss has been shown not only in older adults,^6,7,8,9^ but also after gastric bypass,^10^ and after long-term calorie restriction in younger adults with^11,12^ and without obesity.^13^ Thus, weight loss–induced bone loss likely carries a risk across the lifespan, and identifying treatments that induce clinically relevant weight loss while minimizing bone loss is essential in long-term obesity management.

Glucagon-like peptide-1 receptor agonists (GLP-1RA) are used in obesity management because they induce weight loss via appetite inhibition.^14,15,16^ Physical activity is recommended for healthy weight loss, but most evidence is from prospective cohort studies.^17^ Exercise decreases fat mass and preserves or increases lean mass.^18^ In a recent trial,^19^ 1 year of treatment with the GLP-1RA liraglutide (3.0 mg) or exercise as separate treatments maintained diet-induced weight loss and improved body composition. The combination of exercise and liraglutide was superior in terms of weight reduction and body composition compared with the separate treatments.^19^

Mechanical strain of bone during exercise may increase bone formation^20^ and may preserve bone health during weight loss. Aerobic and resistance exercise during calorie restriction has been shown to counteract bone loss compared with calorie restriction alone.^8,21^ High physical activity is associated with decreased fracture risk in older adults.^22,23^ Other studies have shown no association of exercise with bone health.^24^ The association of exercise with bone health seems type- and intensity-dependent, and heavy resistance or high-impact exercise may be most effective for bone health.^25,26,27^ Notably, the association of exercise after large weight loss with bone health is unknown.

Although a protective effect on bone health has been suggested with GLP-1RA in rodents^28,29^ and human cell lines,^30,31^ evidence of a direct association of GLP-1RA with bone health in humans is limited. Liraglutide (1.2 mg) has been found to increase bone formation and preserve bone mineral content during 1 year of weight maintenance in women with obesity.^12^ In patients with type 2 diabetes, total and hip BMD was unchanged after modest weight loss induced by 1.8 mg of liraglutide.^32,33^ The GLP-1RA exenatide was associated with increased hip BMD and minor weight loss.^34^ Results from meta-analyses on the association of fracture risk with GLP-1RA for patients with diabetes are inconsistent,^35^ where some show slightly decreased^36^ or no difference in fracture risk.^37^ These studies are generally limited by a low number of fractures, no BMD assessments, short duration of intervention, or the use of other diabetic drugs that may have affected fracture risk.

Due to these limitations, the association of GLP-1RA with bone health is not well-established and unknown following larger weight losses and at doses used for obesity treatment. In addition, combining GLP-1RA with exercise has not been investigated in the context of bone health. Thus, the aim of this study was to investigate changes in BMD at clinically relevant sites (hip, lumbar spine, and forearm) in response to diet-induced weight loss followed by 1-year treatment with liraglutide, exercise alone, or both treatments in combination. We hypothesized that combining both treatments would preserve bone mass despite superior long-term weight reduction. Additionally, we tested whether weight maintenance with exercise alone compared with liraglutide elicited different effects on bone health.

## Methods

### Study Design and Setting

This study is a predefined secondary analysis of a randomized clinical trial^19,38^ that was approved by the Regional Ethics Committee in Denmark and the Danish Medicines Agency, and follows the Consolidated Standards of Reporting Trials (CONSORT) reporting guideline for randomized studies. The purpose of the original trial^19,38^ was to investigate exercise alone, liraglutide, and both treatments combined for healthy weight loss maintenance, and the primary outcome was change in body weight (see the study protocol and statistical analysis plan in Supplement 1). The study was conducted from August 2016 to November 2019 at Hvidovre Hospital and the Department of Biomedical Sciences of the University of Copenhagen, Denmark. Participants received oral and written information about the study, and written informed consent was obtained from all participants.

### Participants

Inclusion criteria were age (18-65 years), obesity (body mass index [BMI] of 32-43 [calculated as weight in kilograms divided by height in meters squared]), and safe contraceptive method or postmenopausal status. Sex assigned at birth was self-reported. Exclusion criteria were any known serious chronic illness, including diabetes. For more details, see the trial protocol in Supplement 1.

### Allocation to Intervention

Participants were randomized after the low-calorie diet to 1 of the 4 intervention groups in a 1:1:1:1 ratio stratified by sex and age (<40 years or ≥40 years). Participants, personnel, and investigators were blinded regarding study medication until the primary outcomes were analyzed.^38^ Using a randomization list supplied by Novo Nordisk A/S, a project nurse assigned participants to their respective treatments.

### Interventions

All participants underwent an initial 8-week low-calorie diet of approximately 800 kcal/day. The diet consisted of 4 low-calorie meal replacements per day (Cambridge Weight Plan). Participants who lost at least 5% of their initial body weight were then randomly assigned to a 1-year intervention phase with either exercise and placebo (exercise group), liraglutide (liraglutide group), a combination of exercise and liraglutide (combination group), or placebo (placebo group).

The exercise intervention started with a 6-week ramp-up phase. From week 7 to 52, participants were encouraged to attend group exercise sessions 2 times per week and to perform exercise individually 2 times per week. The group sessions consisted of 30-minute indoor cycling and 15-minute circuit training. The cycling was interval-based and of vigorous intensity, aimed at an average intensity of 80% maximal heart rate or greater. The circuit training involved a combination of vigorous-intensity aerobic exercise and muscle-strengthening exercises using body weight or external resistance. Typically, 3 circuits of 5 exercises were performed for 40 seconds interspersed with 20-second breaks. The type of exercise performed at individual sessions was of the participants’ own choice but was recommended to be of moderate-to-vigorous intensity. The most frequently performed individual exercises were cycling, running, brisk walking, and individual circuit training. Participants wore heart rate monitors during all exercise sessions to monitor adherence. Those not randomized to exercise were instructed to maintain usual physical activity throughout the trial. The full description of the exercise intervention with examples of exercise programs is available in the original article.^19^

Liraglutide or volume-matched placebo was administered as once daily subcutaneous injections in the abdomen. The starting dose was 0.6 mg/day with weekly increments of 0.6 mg until reaching a dose of 3.0 mg/day. Participants who did not tolerate 3.0 mg/day received the highest tolerated dose. All participants attended weight consultations with dietetic support aligned with the dietary recommendations provided by the Danish Authorities^39^ approximately once a month during the intervention phase.

### Outcomes

#### Body Weight and Body Composition

Body weight and body composition were measured in fasting state, and changes in these outcomes from week 0 to 52 have been published previously.^19^ Fat mass and lean mass were measured with dual-energy x-ray absorptiometry (DXA), and body fat percentage was calculated as fat mass divided by body weight multiplied by 100.

#### BMD

BMD in grams per centimeter squared was measured with DXA in fasting state separately at the left hip and lumbar spine (L1-L4), which are considered the important sites for assessments of fracture risk and treatment response, respectively.^40,41^ Distal forearm (1/3 radius and ulna) and whole-body BMD were also assessed. DXA-derived BMD measurements are recommended to be interspersed by periods of 4 to 6 months or greater.^42,43^ Therefore, changes in BMD were calculated from before the low-calorie diet to 1 year after randomization.

#### Markers of Bone Turnover

Fasting blood samples were collected after an overnight fast to measure plasma type 1 collagen cross-linked C-telopeptide (P-CTX), a marker of bone resorption, and plasma propeptide of type 1 procollagen (P-P1NP), a marker of bone formation, by a fully automated immunoassay system using IDS-iSYS CTX and IDS-iSYS intact serum procollagen type I N-propeptide assays (Immunodiagnostic Systems). Both assays are chemiluminescence methods.

### Sample Size Estimation

The sample size estimation was based on change in body weight.^19^ It was estimated that at least 30 completers in each group would be required to detect a 4.0-kg difference (power = 0.80; 2-sided α = .05) between any of the 4 groups.

### Statistical Analysis

Changes in outcomes were analyzed in the intention-to-treat population (all randomized participants) using constrained linear mixed models with inherent baseline (week −8) adjustment.^44,45^ The model was specified with time, a time-treatment interaction, sex, and age (<40 years or ≥40 years) as fixed effects and an unstructured covariance pattern to account for repeated measurements for each participant. Missing data was assumed to be missing at random and was implicitly handled by maximum likelihood estimation in the mixed model. Within-group changes from week −8 to 52 and between-group differences at week 52 with adjustment for covariates and potential differences at baseline (week −8) were presented as estimated means (95% CI). Hip and lumbar spine BMD were chosen as the main outcomes because they are the most frequently used sites in the clinical assessment of osteoporosis and are preferred for assessments of fracture risk and treatment response.^40,41,46^ Thus, between-group differences in hip and lumbar spine BMD were null hypothesis tested and presented with *P* values. A 2-sided *P* < .05 was considered statistically significant. Adequacy of model assumptions were evaluated with graphical methods. For data that did not meet the model assumptions, analyses were performed on log-transformed data. For presentation, log-transformed outcomes were back-transformed and presented as estimated geometric means (95% CIs). Statistical analyses were performed in SAS Enterprise Guide version 8.1 (SAS Institute Inc). Data analysis was conducted from March to April 2023, with additional analysis in February 2024 during revision.

## Results

### Study Population

A total of 195 participants (mean [SD] age, 42.84 [11.87] years; 124 female [64%] and 71 male [36%]; mean [SD] BMI, 37.00 [2.92]) completed the low-calorie diet and were randomized to the exercise group (48 participants), liraglutide group (49 participants), combination group (49 participants), or placebo group (49 participants). BMD measurements were available at week 52 for 158 participants (81%) to 161 participants (83%) depending on the site (eFigure 1 in Supplement 2). Characteristics of participants at inclusion are shown in Table 1. No participants received any medications for osteoporosis, and no fragility fractures were reported in any group. Safety outcomes have been reported elsewhere.^19^

**Table 1.** Characteristics of Participants Before Low-Calorie Diet^a^

Characteristic	Participants, No (%) (N = 195)			
	Placebo group (n = 49)	Exercise group (n = 48)	Liraglutide group (n = 49)	Combination group (n = 49)
Age, mean (SD), y	42.95 (11.54)	42.97 (12.37)	43.23 (11.64)	42.20 (12.28)
Sex^b^				
Female	31 (65)	31 (63)	31 (65)	31 (65)
Male	18 (35)	17 (37)	18 (35)	18 (35)
Menopause	6 (12)	6 (13)	5 (10)	8 (16)
Regular smoking	7 (14)	7 (15)	6 (12)	4 (8)
Vitamin D supplements^c^	6 (14)	7 (17)	4 (9)	6 (14)
Body weight, mean (SD), kg	109.69 (14.78)	109.96 (14.63)	107.63 (15.66)	111.89 (14.56)
Body mass index, mean (SD)^d^	36.52 (2.92)	37.09 (3.09)	36.97 (3.25)	37.24 (2.42)
Whole-body BMD, mean (SD), g/cm^2^	1.25 (0.10)	1.26 (0.10)	1.24 (0.11)	1.23 (0.11)
Whole-body T score, mean (SD)	0.59 (0.80)	0.74 (0.92)	0.48 (0.86)	0.37 (0.86)
Total hip BMD, mean (SD), g/cm^2^	1.07 (0.13)	1.09 (0.13)	1.03 (0.11)	1.05 (0.10)
Total hip T score, mean (SD)	1.27 (1.08)	1.45 (1.11)	0.95 (0.83)	1.05 (0.85)
Lumbar spine BMD, mean (SD), g/cm^2^	1.12 (0.13)	1.14 (0.16)	1.09 (0.14)	1.10 (0.13)
Lumbar spine T score, mean (SD)	0.75 (1.25)	0.97 (1.58)	0.45 (1.40)	0.52 (1.25)
Distal forearm BMD, mean (SD), g/cm^2^	0.76 (0.09)	0.75 (0.08)	0.75 (0.10)	0.75 (0.08)
Plasma CTX, median (IQR), ng/L	271.2 (205.7-415.7)	231.4 (178.1-335.9)	305.0 (210.6-388.5)	248.7 (173.9-385.0)
Plasma P1NP, median (IQR), μg/L	52.8 (45.9-71.8)	50.7 (39.5-60.9)	55.3 (41.1-67.9)	55.8 (41.4-72.4)
Plasma parathyrin, median (IQR), pmol/L	5.5 (4.5-6.8)	5.4 (4.5-6.9)	5.3 (4.7-6.5)	5.6 (4.6-6.8)
Plasma calcium, mean (SD), mg/dL	9.40 (0.36)	9.32 (0.32)	9.28 (0.28)	9.24 (0.28)
Plasma 25-hydroxovitamin D (D2 + D3), mean (SD), ng/mL	23.78 (10.69)	21.54 (10.18)	22.15 (8.63)	23.50 (8.56)

Abbreviations: BMD, bone mineral density; CTX, type 1 collagen cross-linked C-telopeptide; P1NP, propeptide of type 1 procollagen.

SI Conversions: To convert calcium to millimoles per liter, multiply by 0.25; plasma 25-hydroxovitamin D to nanomoles per liter, multiply by 2.496.

^a^ Values are at inclusion (before the low-calorie diet). Participants were randomized after the low-calorie diet.

^b^ Sex assigned at birth.

^c^ Participants consuming dietary vitamin D supplements or multivitamins containing vitamin D at inclusion (before the low-calorie diet).

^d^ Body mass index was calculated as weight in kilograms divided by height in meters squared.

### Compliance

As previously reported,^19^ the mean (SD) exercise volume was 118 (74) minutes/week at a mean (SD) intensity of 78% (4%) of maximum heart rate in the exercise group and 111 (73) minutes/week at 79% (5%) of maximum heart rate in the combination group. Eight participants completed the study at a lower medication dose (5 participants [12%] in the liraglutide group and 3 participants [7%] in the combination group). A total of 11 participants completed the study but discontinued study medication (1 in the placebo group, 7 in the exercise group, 1 in the liraglutide group, and 2 in the combination group).^19^

### Body Weight and Body Composition

**Figure 1.**
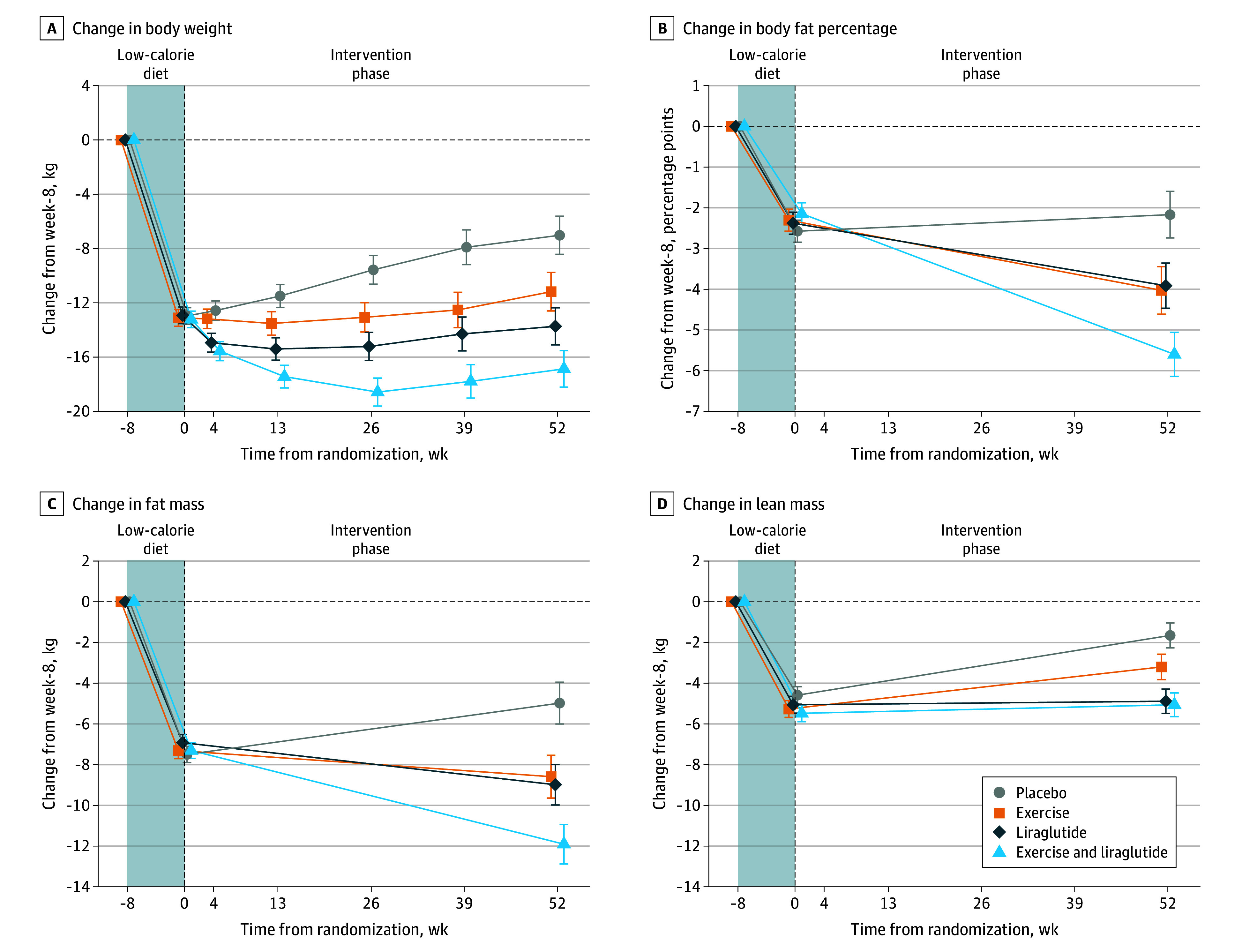
Changes in Body Weight and Body Composition During the Study Changes are shown for body weight (A), body fat percentage (B), fat mass (C), and lean mass (D). Values are estimated mean changes in kilograms after a low-calorie diet (weeks −8 to 0) and 52 weeks after randomization to treatment with placebo, exercise, liraglutide, or the combination of exercise and liraglutide. The shaded area indicates the period of the low-calorie diet. Results are from the intention-to-treat population (all randomized participants). Randomization was done at week 0, immediately after the low-calorie diet. Error bars are standard errors of the mean.

Total estimated mean change in weight loss during the study was 7.03 kg (95% CI, 4.25-9.80 kg) in the placebo group, 11.19 kg (95% CI, 8.40-13.99 kg) in the exercise group, 13.74 kg (95% CI, 11.04-16.44 kg) in the liraglutide group, and 16.88 kg (95% CI, 14.23-19.54 kg) in the combination group. After a low-calorie diet-induced weight loss of 13.1 kg for the overall cohort, the placebo group regained weight, the exercise and liraglutide groups maintained weight loss, and the combination group lost additional weight (Figure 1A). After the total study period (8-week low-calorie diet and 1-year intervention phase), the exercise and liraglutide groups showed reduced fat percentage and fat mass, with additive reductions in the combination group (Figure 1B and C). The exercise group had increased lean mass during the 1-year intervention period (Figure 1D). The combination group preserved lean mass despite the largest fat mass reduction.

### Bone Mineral Density

**Figure 2.**
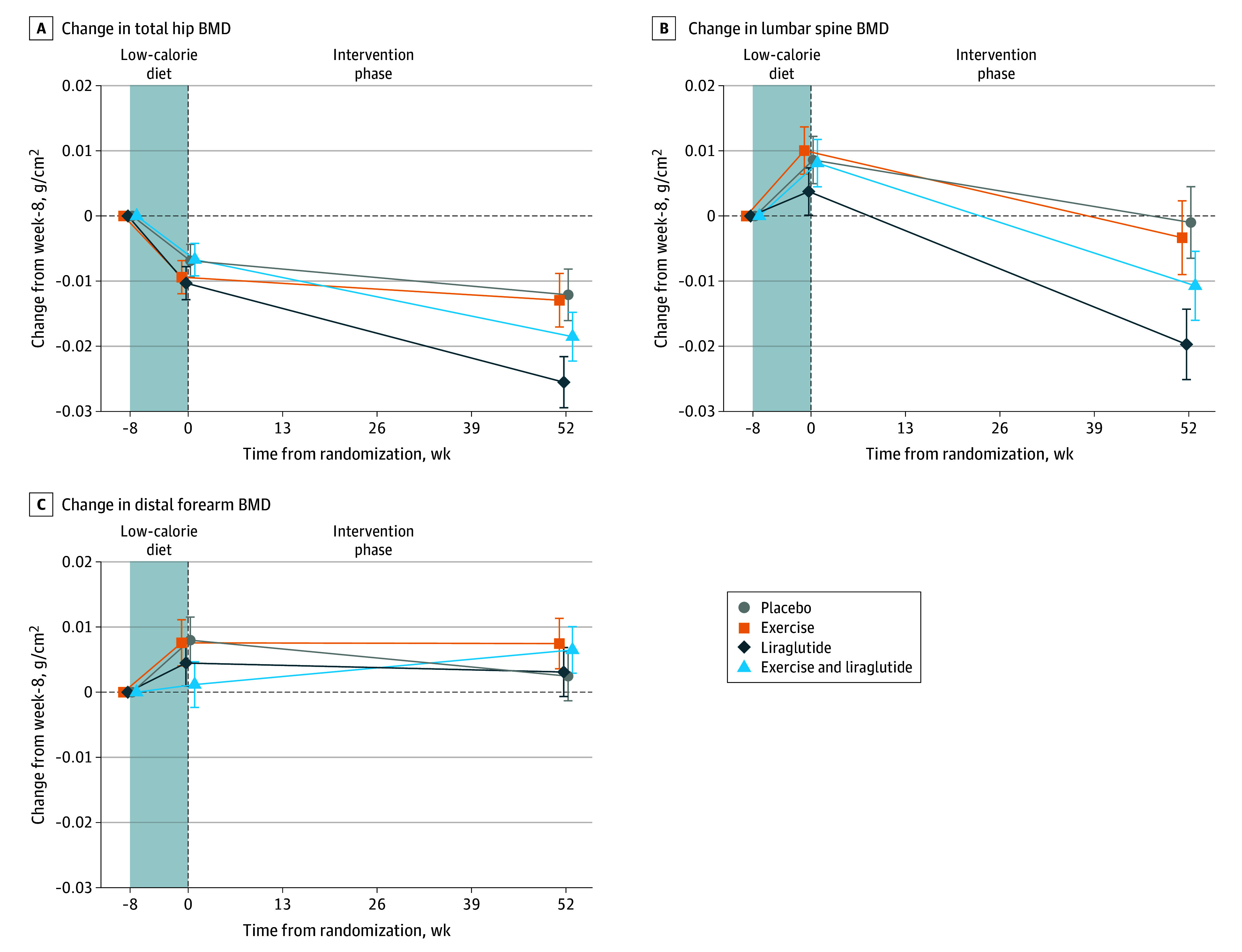
Changes in Bone Mineral Density (BMD) During the Study Changes are shown for BMD at the total hip (A), lumbar spine (B), and distal forearm (C). Values are estimated mean changes in BMD after a low-calorie diet (weeks −8 to 0) and 52 weeks after randomization to treatment with placebo, exercise, liraglutide, or the combination of exercise and liraglutide. The shaded area indicates the period of the low-calorie diet. Results are from the intention-to-treat population (all randomized participants). Randomization was done at week 0, immediately after the low-calorie diet. Error bars are standard errors of the mean.

Changes in BMD from week −8 (before low-calorie diet) to week 52 are shown in Figure 2 and Table 2. In the combination group, BMD was unchanged compared with the placebo group at the hip (mean change, −0.006 g/cm^2^; 95% CI, −0.017 to 0.004 g/cm^2^; *P* = .24) and lumbar spine (mean change, −0.010 g/cm^2^; 95% CI, −0.025 to 0.005 g/cm^2^; *P* = .20) (Figure 2A and B). In the liraglutide group, hip BMD decreased compared with the exercise group (mean change, −0.013 g/cm^2^; 95% CI, −0.024 to −0.001 g/cm^2^; *P* = .03) and placebo group (mean change, −0.013 g/cm^2^; 95% CI, −0.024 to −0.002 g/cm^2^; *P* = .02). Likewise, in the liraglutide group, lumbar spine BMD decreased compared with the exercise group (mean change, −0.016 g/cm^2^; 95% CI, −0.032 to −0.001g/cm^2^; *P* = .04) and placebo group (mean change, −0.019 g/cm^2^; 95% CI, −0.034 to 0.002 g/cm^2^; *P* = .02). Distal forearm BMD increased in the exercise and combination groups, with no differences between the 4 groups regarding distal forearm BMD (Figure 2C). Subgroup analyses according to sex (male and female) and age (<40 years or ≥40 years) are available for BMD outcomes in the eTable in Supplement 2. Compared with placebo, the liraglutide and combination treatments were associated with increased whole-body BMD (eFigure 2 in Supplement 2).

**Table 2.** Change in Site-Specific Bone Mineral Density

End point	Change in bone mineral density, g/cm^2^, mean (95% CI)^a^			
	Placebo group	Exercise group	Liraglutide group	Combination group
Total hip				
Within-group change	−0.012 (−0.020 to −0.004)	−0.013 (−0.021 to −0.005)	−0.026 (−0.033 to −0.018)	−0.019 (−0.026 to −0.011)
Difference vs placebo	NA	−0.001 (−0.012 to 0.010)	−0.013 (−0.024 to −0.002)	−0.006 (−0.017 to 0.004)
Difference vs exercise	NA	NA	−0.013 (−0.024 to −0.001)	−0.006 (−0.017 to 0.005)
Difference vs liraglutide	NA	NA	NA	0.007 (−.004 to 0.018)
Lumbar spine	NA	NA	NA	NA
Within-group change	−0.001 (−0.012 to 0.010)	−0.003 (−0.015 to 0.008)	−0.020 (−0.030 to −0.009)	−0.011 (−0.021 to 0.000)
Difference vs placebo	NA	−0.002 (−0.018 to 0.013)	−0.019 (−0.034 to −0.004)	−0.010 (−0.025 to 0.005)
Difference vs exercise	NA	NA	−0.016 (−0.032 to −0.001)	−0.007 (−0.023 to 0.008)
Difference vs liraglutide	NA	NA	NA	0.009 (−0.006 to 0.024)
Distal forearm				
Within-group change	0.003 (−.005 to 0.010)	0.008 (0.000 to 0.015)	0.003 (−.004 to 0.011)	0.007 (0.000 to 0.014)
Difference vs placebo	NA	0.005 (−0.006 to 0.016)	0.001 (−0.010 to 0.011)	0.004 (−0.006 to 0.014)
Difference vs exercise	NA	NA	−0.004 (−0.015 to 0.006)	−0.001 (−0.011 to 0.009)
Difference vs liraglutide	NA	NA	NA	0.003 (−0.007 to 0.014)

^a^ Changes and differences are estimated means (95% CI) from before 8 weeks of low-calorie diet (week −8) to 52 weeks after randomization (week 0) to treatment for 1 year.

### Bone Turnover Markers

**Figure 3.**
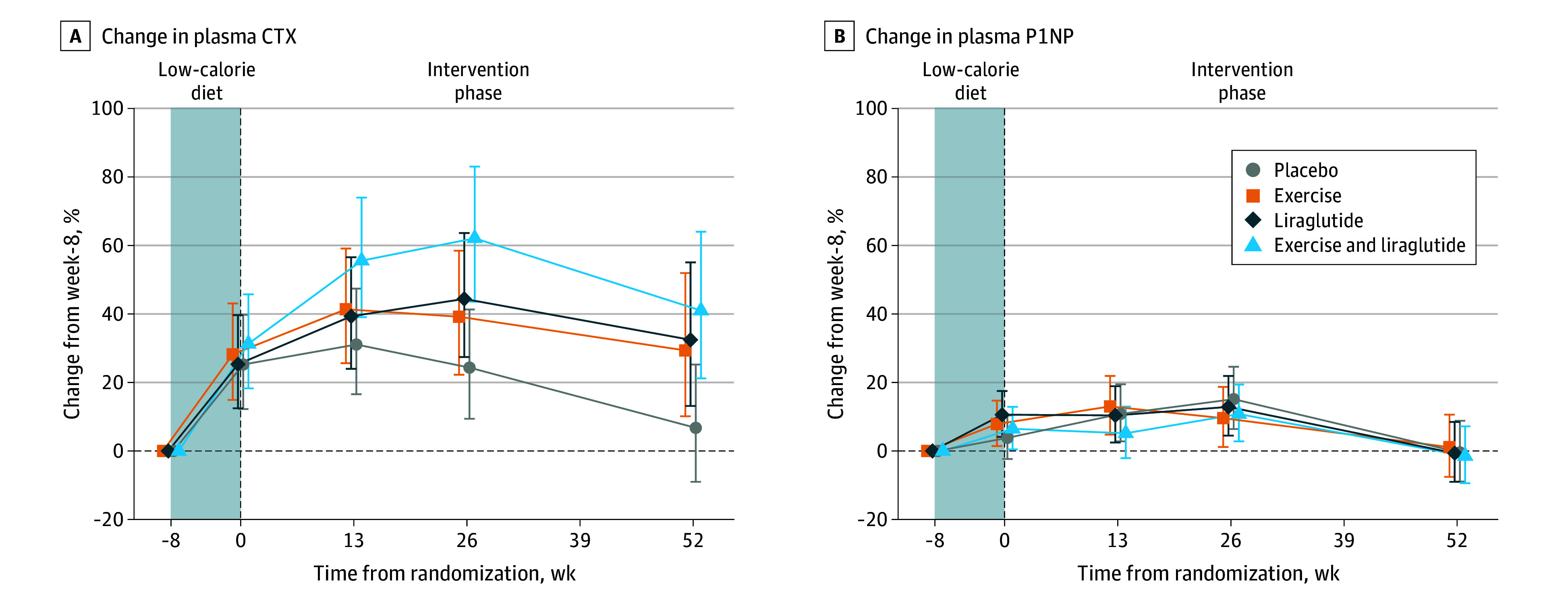
Changes in Markers of Bone Turnover During the Study Changes are shown for plasma type 1 collagen cross-linked C-telopeptide (CTX; A) and plasma propeptide of type 1 procollagen (P1NP; B) during the study. These outcomes were initially analyzed on a log scale. For presentation, these outcomes were back-transformed and expressed as percentage change from week −8 (calculated as estimated ratio − 1 × 100) with 95% CIs. Results are for participants in the intention-to-treat population (all participants who were randomized at week 0). The shaded areas indicate the low-calorie diet period.

In response to the 8-week low-calorie diet, P-CTX increased by a mean of 27% (95% CI, 21%-35%) (Figure 3A). At week 26, P-CTX decreased again in all treatment groups, with the lowest levels in the placebo group. P-P1NP increased by a mean of 7% (95% CI, 4%-10%) during the low-calorie diet (Figure 3B). At week 52, P1NP had returned to the initial levels in all 4 groups.

## Discussion

This secondary analysis of a randomized clinical trial aimed to investigate changes in site-specific BMD during a diet-induced weight loss followed by a 1-year intervention with either liraglutide (3.0 mg/day), exercise, or the combination of both, compared with placebo. The combination treatment led to the largest weight and body fat reduction while preserving hip, spine, and forearm BMD levels compared with the placebo group. Liraglutide alone led to weight and fat loss compared with placebo; however, this was associated with decreased hip and spine BMD compared with placebo and exercise alone. Exercise alone led to similar weight loss as liraglutide alone but with increased lean mass and preserved hip and spine BMD. Collectively, our results show that the combination of exercise and GLP-1RA was the most effective weight loss strategy while preserving bone health.

Despite a substantial weight loss of 16.9 kg for the combination group vs 7.0 kg for the placebo group, the combination treatment preserved site-specific BMD compared with placebo. Thus, in the combination group, the preserved bone mass was observed despite a weight reduction of a magnitude that is clinically relevant in the context of novel incretin-based obesity therapies, such as semaglutide and tirzepatide, which resulted in weight losses of around 15% to 19% in a systematic review.^47^

Liraglutide without exercise was also effective by maintaining a 13.7 kg weight loss after 1 year, which was 6.4 kg more than the placebo group. However, in contrast with exercise and liraglutide combined, liraglutide alone led to a decrease in hip and spine BMD compared with placebo. Thus, after weight reduction, adding exercise to long-term GLP-1RA treatment preserves bone at clinically important sites of fracture risk (ie, the hip and spine). Additionally, exercise and combination treatment increased forearm BMD, which is independent of weight-carrying activities. Exercise has also been shown to preserve BMD in persons who have undergone gastric bypass surgery and are at increased risk of bone fractures,^48^ collectively highlighting that exercise should be considered alongside GLP-1-based therapy to minimize bone loss. In our study, liraglutide alone compared with exercise alone, reduced hip BMD despite similar weight loss. The exercise and liraglutide groups had similar reductions in body fat percentage, but this was associated with increased lean mass and decreased fat mass with exercise in contrast with only fat mass loss with liraglutide. The magnitude of the association of bone mass with lean mass is greater than the association of bone mass than total body mass.^49,50^ Thus, preserved or increased lean mass could be a means by which exercise can preserve bone mass during obesity treatment.

The liraglutide and combination treatments were associated with increased whole-body BMD. In agreement, 1.2 mg of liraglutide was shown to prevent a weight loss–induced decrease in whole-body bone mineral content during 1 year after diet-induced weight loss in women with obesity.^12^ The finding that whole-body BMD, mostly reflecting cortical bone mass,^51^ increased with liraglutide, is in line with the observation of cortical osteopenia and fragility in GLP-1 receptor knockout mice.^52,53^ Interestingly, in the present study, exercise alone and in combination with liraglutide was associated with mostly preserved cortical bones (forearm) and more trabecular bones (hip and spine). Because fractures at the hip and spine are associated with increased mortality risk,^4,54^ the exercise-associated preservation of site-specific BMD is of clinical relevance.

The low-calorie diet increased P-CTX, the marker of bone resorption, by 27% and P-P1NP, the marker of bone formation, by 7%. These changes are consistent with results from a similar low-calorie diet intervention^12^ and indicate increased bone turnover in favor of bone resorption in response to diet-induced weight loss. Elevated bone resorption during the low-calorie diet may point to caloric restriction as a driver of bone loss. The steepest increases in P-CTX occurred during the low-calorie diet, and the levels waned during the last part of the intervention phase. From week 26, P-CTX decreased again in all treatment groups, reflecting more stable body weight development. No changes in P-P1NP were observed in any active treatment group compared with placebo, suggesting that bone formation was not suppressed despite the concomitant weight reductions.

Strengths of the study include the randomized design and that changes in bone health were assessed at sites of clinical relevance for fragility fractures (ie, the hip, lumbar spine, and distal forearm). To our knowledge, this is the first study to assess the effect of GLP-1RA and exercise on bone health both as separate treatments and in combination. The mean weight loss obtained in our study was comparable to the marked weight losses obtained with novel GLP-1RA therapies and is, therefore, clinically relevant in the context of contemporary obesity treatment.^55,56^

### Limitations

This study has limitations. Because we included adults aged 18 to 65 years without other chronic diseases, the results may not be generalizable to patients with diabetes or older individuals (ie, populations at increased risk of bone fractures). However, the observation that BMD decreased with liraglutide alone but not in combination with exercise, supports the use of exercise with obesity medications in populations with decreased bone mass (eg, after menopause). The sample size was calculated based on changes in body weight and bone health was a prespecified secondary outcome. However, sample size was large compared with most previous randomized clinical trials, which have investigated the effects of GLP-1RA or exercise on bone health.^57^ Because change in bone health was a secondary outcome, the analyses were not adjusted for multiple comparisons and should be considered exploratory. The study was conducted in Denmark with an ancestrally homogenous study population. The study sample was heterogeneous, including males and premenopausal and postmenopausal females. While this heterogeneity may have increased variation, it also improves the generalizability of the findings.

## Conclusions

In this randomized clinical trial, the combination of exercise and liraglutide was the most effective weight loss strategy while preserving bone health. Despite similar weight loss, liraglutide treatment reduced hip and spine BMD compared with exercise alone. Our findings highlight the importance of combining exercise with GLP-1 RA treatment for bone health.

## Notes

Supplement 1.
Trial Protocol and Statistical Analysis Plan

Supplement 2. **eFigure 1.** Study Flow Chart **eTable.** Change in Site-Specific Bone Mineral Density From Week −8 to 52 in Subgroups **eFigure 2.** Changes in Whole-Body Bone Mineral Density During the Study

Supplement 3.
Data Sharing Statement
