# Supplementary material for: Bone Health After Exercise Alone, GLP-1 Receptor Agonist Treatment, or Combination Treatment: A Secondary Analysis of a Randomized Clinical Trial
Source: JAMA Netw Open. 2024 Jun 25;7(6):e2416775. doi: 10.1001/jamanetworkopen.2024.16775 (PMC11200146; doi:10.1001/jamanetworkopen.2024.16775)
Supplement: Supplement 2. — eFigure 1. Study Flow Chart eTable. Change in Site-Specific Bone Mineral Density From Week −8 to 52 in Subgroups eFigure 2. Changes in Whole-Body Bone Mineral Density During the Study [file jamanetwopen-e2416775-s002.pdf]

Supplemental Online Content

Jensen SBK, Sørensen V, Sandsdal RM, et al. Bone health after exercise alone, GLP-1 receptor agonist treatment, or combination treatment: a secondary analysis of a randomized clinical trial. *JAMA Netw Open*.
2024;7(6):e2416775. doi:10.1001/jamanetworkopen.2024.16775

eFigure 1. Study Flow Chart

eTable. Change in Site-Specific Bone Mineral Density From Week −8 to 52 in Subgroups

eFigure2. Changes in Whole-Body Bone Mineral Density During the Study

This supplemental material has been provided by the authors to give readers additional information about their work.

© 2024 Jensen SBK et al. *JAMA Network Open.*

eFigure 1. Study Flow Chart

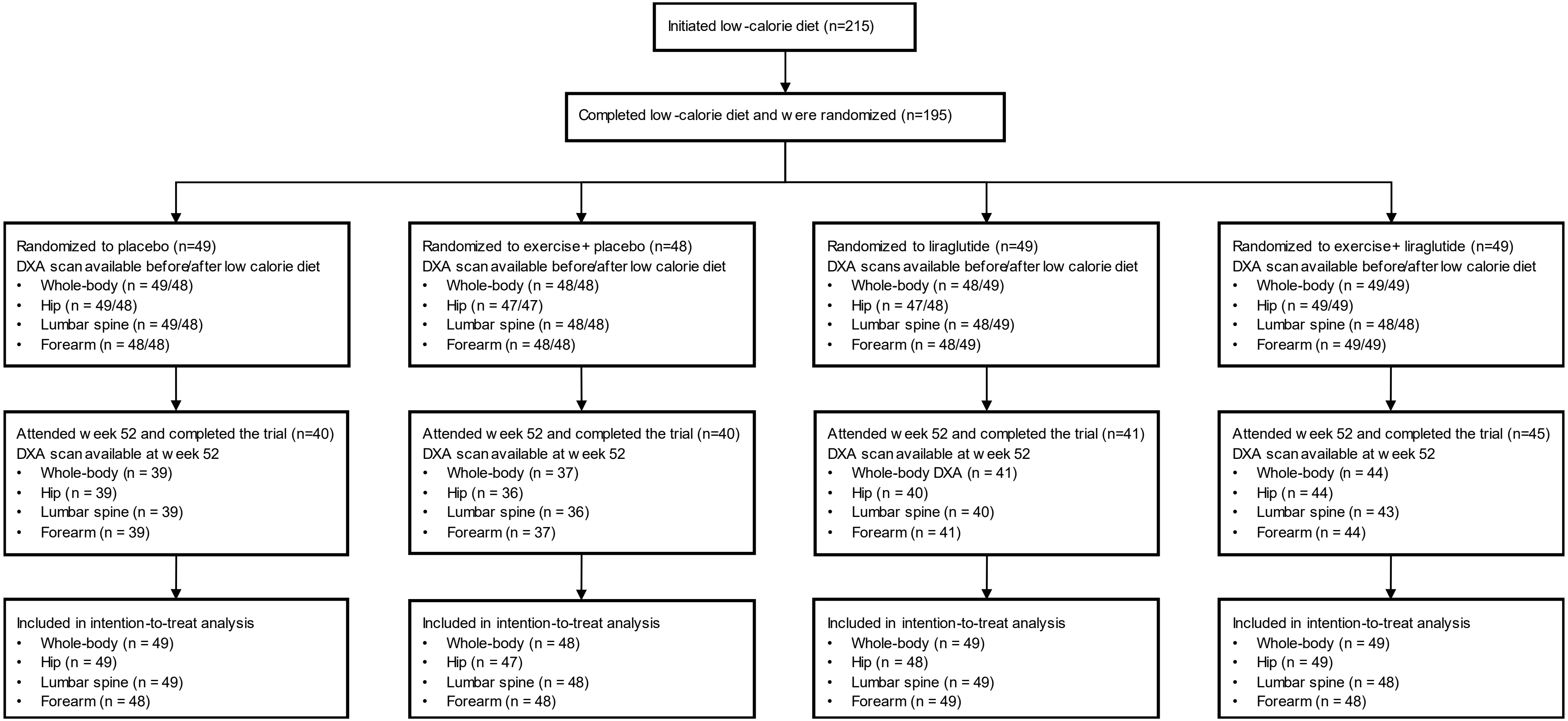

© 2024 Jensen SBK et al. *JAMA Network Open.*

eTable. Change in site-Specific Bone Mineral Density From Week -8 to 52 in Subgroups

	Placebo group	Exercise group	Liraglutide group	Combination group
	**Mean (95% CI)**	**Mean (95% CI)**	**Mean (95% CI)**	**Mean (95% CI)**
**Total hip BMD (g/cm^2)**				
Full analysis set	-.012 (-.020; -.004)	-.013 (-.021; -.005)	-.026 (-.033; -.018)	-.019 (-.026; -.011)
Sex				
Males (n=85)	-.011 (-.022; -.000)	-.010 (-.021; .002)	-.024 (-.036; -.012)	-0.012 (-0.022; -0.001)
Females (n=137)	-.013 (-.024; -.002)	-.015 (-.027; -.004)	-.026 (-.036; -.015)	-0.023 (-0.033; -0.013)
Age				
≥40 years (n=125)	-.015 (-.026; -.005)	-.015 (-.025; -.005)	-.026 (-.036; -.016)	-.022 (-.032; -.012)
<40 years (n=97)	-.006 (-.018; .007)	-.008 (-.023; .006)	-.026 (-.039; -.012)[a]	-.013 (-.025; -.001)
**Lumbar spine BMD (g/cm^2)**				
Full analysis set	-.001 (-.012; .010)	-.003 (-.015; .008)	-.020 (-.030; -.009)	-.011 (-.021; .000)
Sex				
Males (n=85)	.013 (-.007; .033)	.005 (-.015; .026)	-.023 (-.044; -.003)[a,b]	-.007 (-.026; .013)
Females (n=137)	-.010 (-.022; .003)	-.009 (-.022; .004)	-.017 (-.029; -.005)	-.014 (-.026; -.001)
Age				
≥40 years (n=125)	-.007 (-.021; .007)	-.001 (-.015; .012)	-.026 (-.04; -.013)[a,b]	-.021 (-.035; -.007)[b]
<40 years (n=97)	.009 (-.007; .026)	-.007 (-.025; .012)	-.008 (-.026; .009)	.005 (-.011; .021)
**Distal forearm BMD (g/cm^2)**				
Full analysis set	.003 (-.005; .010)	.008 (.000; .015)	.003 (-.004; .011)	.007 (.000; .014)
Sex				
Males (n=85)	-.001 (-.014; .013)	.007 (-.006; .021)	.009 (-.005; .023)	.000 (-.013; .013)
Females (n=137)	.004 (-.005; .013)	.009 (-.001; .018)	-.001 (-.01; .007)	.011 (.002; .020)
Age				
≥40 years (n=125)	-.002 (-.012; .008)	.009 (-.001; .019)	.002 (-.008; .012)	.009 (.000; .019)
<40 years (n=97)	.011 (.000; .021)	.002 (-.010; .014)	.006 (-.006; .017)	.001 (-.009; .011)

Changes/differences are estimated means (95% CI) from before eight weeks of low-calorie diet (week -8) to 52 weeks after randomization (week 0) to treatment for one year.
[a] difference from the placebo group
[b] difference from the exercise group
BMD, bone mineral density.

© 2024 Jensen SBK et al. *JAMA Network Open.*

eFigure 2. Changes in Whole-Body Bone Mineral Density During the Study

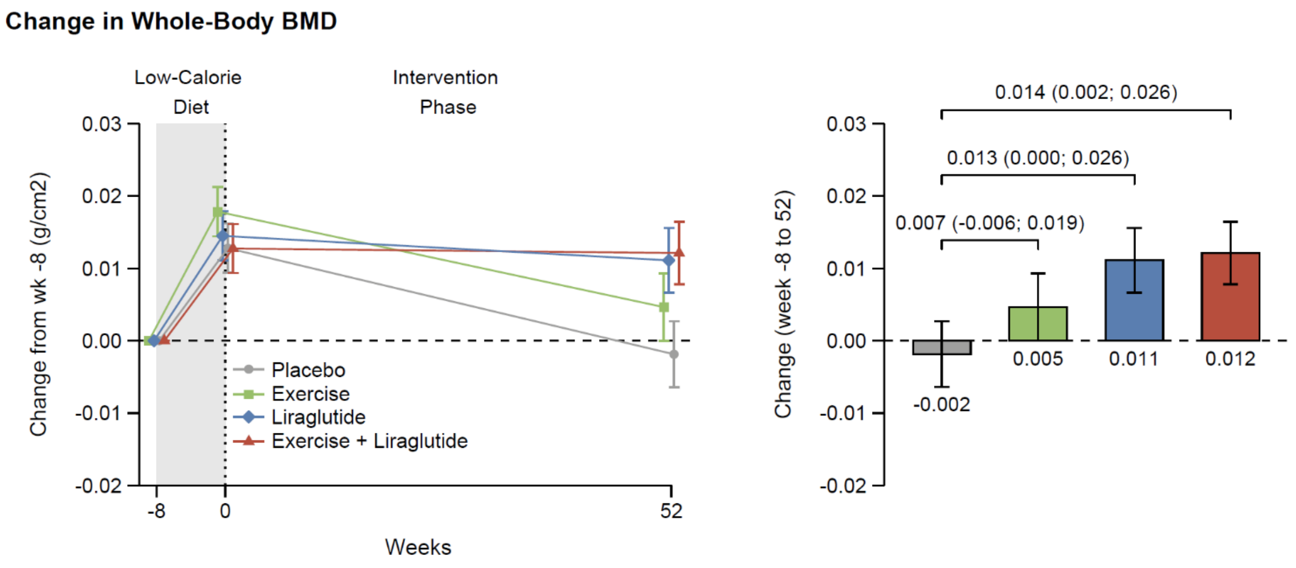

The line graph on the left shows the estimated mean changes in whole-body bone mineral density (BMD) after a low-calorie diet (week -8 to 0) and 52 weeks after randomization to treatment with placebo, exercise, liraglutide, or the combination of exercise and liraglutide. The grey shaded area indicates the period of the low-calorie diet. The bar graph on the right shows the mean changes in whole-body BMD from before the low-calorie diet (week -8) to week 52 with estimated mean differences and 95% confidence intervals between placebo and the active treatments. Results are from the intention-to-treat population (all randomized participants). Randomization was done at week 0, immediately after the low-calorie diet. Error bars are SE of the mean.

© 2024 Jensen SBK et al. *JAMA Network Open.*
