# Supplementary material for: Bone Health After Exercise Alone, GLP-1 Receptor Agonist Treatment, or Combination Treatment: A Secondary Analysis of a Randomized Clinical Trial
Source: JAMA Netw Open. 2024 Jun 25;7(6):e2416775. doi: 10.1001/jamanetworkopen.2024.16775 (PMC11200146; doi:10.1001/jamanetworkopen.2024.16775)
Supplement: Supplement 3. — Data Sharing Statement [file jamanetwopen-e2416775-s003.pdf]

Data Sharing Statement

Jensen. BoneHealth After Exercise Alone, GLP-1 Receptor Agonist Treatment, or Combination Treatment. *JAMA Netw Open*. Published June 25, 2024.
doi:10.1001/jamanetworkopen.2024.16775

Data
Data available: Yes
Data types: Deidentified participant data
How to access data: De-identified data under the General Data Protection Regulations (GDPR) and local regulations of Denmark may be available for research collaboration purposes upon reasonable request to the corresponding author (Signe Sørensen Torekov, torekov@sund.ku.dk) and will require the approval of the corresponding author and the completion of a data processing agreement.
When available: With publication

Supporting Documents
Document types: None

Additional Information
Who can access the data: De-identified data under the General Data Protection Regulations (GDPR) and local regulations of Denmark may be available for research collaboration purposes upon reasonable request to the corresponding author (Signe Sørensen Torekov, torekov@sund.ku.dk) and will require the approval of the corresponding author and the completion of a data processing agreement.
Types of analyses: De-identified data under the General Data Protection Regulations (GDPR) and local regulations of Denmark may be available for research collaboration purposes upon reasonable request to the corresponding author (Signe Sørensen Torekov, torekov@sund.ku.dk) and will require the approval of the corresponding author and the completion of a data processing agreement.
Mechanisms of data availability: De-identified data under the General Data Protection Regulations (GDPR) and local regulations of Denmark may be available for research collaboration purposes upon reasonable request to the corresponding author (Signe Sørensen Torekov, torekov@sund.ku.dk) and will require the approval of the corresponding author and the completion of a data processing agreement.
